# Emotional and behavioral problems in adolescents in the context of COVID-19: a mixed method study*

**DOI:** 10.1590/1518-8345.6273.3744

**Published:** 2022-11-07

**Authors:** Carolina Ferreira Peterle, Caroline Lima Fonseca, Bruna Hinnah Borges Martins de Freitas, Maria Aparecida Munhos Gaíva, Paula Manuela Jorge Diogo, Juliano Bortolini

**Affiliations:** 1 Universidade Federal de Mato Grosso, Faculdade de Enfermagem, Cuiabá, MT, Brazil.; 3 Escola Superior de Enfermagem de Lisboa, Departamento de Enfermagem da Criança e do Jovem, Lisboa, Portugal.; 4 Universidade Federal de Mato Grosso, Departamento de Estatística, Cuiabá, MT, Brazil.

**Keywords:** Emotions, Behavior, Mental Health, Adolescent, COVID-19 Pandemic, Nursing

## Abstract

**Objective:**

to estimate the prevalence of emotional and behavioral problems in adolescents in the context of the COVID-19 pandemic e to explore adolescents’ perception of emotional and behavioral problems identified.

**Method:**

mixed-method explanatory sequential design. Participants were 479 adolescents aged 15 to 18 from a Brazilian Central-West region capital.

**Results:**

with a total of 479 participants, mean age was 16.03 years (SD=1.01). The prevalence of emotional and behavioral problems was 61.17%, and there was a difference between the sexes (ORb=2.93; p<0.01). The highest prevalence was related to peer relationship problems (54.49%) and emotional symptoms (52.40%). Adolescents noticed an increase in loneliness, anxiety, sadness, distancing from friends and difficulties in socializing during the pandemic.

**Conclusion:**

most of the investigated adolescents were classified as having emotional and behavioral problems, and girls were more likely to have them than boys. The adolescents’ statements reinforce the quantitative findings. In this way, there is a need to implement actions to promote and restore the adolescents’ mental health, in order to mitigate the COVID-19 pandemic socio-emotional impact on this population.

## Introduction

Adolescence is characterized by the transition between childhood and adulthood, and it is a process marked by several physical, psychological, and social transformations, which are essential for adolescents to mature and be able to live independently^1^. There is still no consensus regarding the age limits of this phase among scholars in the area, but according to the World Health Organization (WHO)^2^, adolescence is the phase from 10 to 19 years of age, and young adolescents are those aged between 15 and 19 years. However, according to the 1990 Brazilian Child and Adolescent Statute, adolescence includes the age group from 12 to 18 years.

Adolescents experience several psychological, cognitive, social, sexual, and moral changes, which relate to each other in a timeless way and are influenced by physical development (puberty), and the search for personal identity and autonomy^3^. The author also emphasizes the relevance of psychosocial development at this phase, with the improvement of emotional and social skills - essential for well-being -, such as the ability to deal with emotions and to relate affectively with other people.

In this context, experiencing emotional distress in adolescence can trigger aggressive and depressive symptoms, which are harmful to the adolescents’ physical and mental health, in addition to compromising social, cognitive, and academic functioning^4^. Among the events and circumstances capable of triggering emotional distress in adolescents, the COVID-19 pandemic stands out, which was identified in December 2019 in Wuhan, Hubei province, China, characterized by an acute infectious pneumonia^5^. 

In a short period of time, a virus called SARS-CoV-2 spread around the world due to its high transmissibility, causing the coronavirus disease^5^. From then on, the WHO began to recommend limiting physical contact in order to reduce transmission and, therefore, an increase in cases, indicating the adoption of domestic quarantine and physical distancing as control strategies^6^. Consequently, there was a loss of intimate and social contact, culminating in greater moments of stress with feelings of loneliness and/or anger^7^ ^-^ ^8^, compromising the adolescents’ emotions and behaviors.

Emotional and behavioral problems (EBP) are characterized by generalized depressed mood, the inability to establish satisfactory interpersonal relationships, behaviors or feelings considered inappropriate in certain circumstances, and a tendency to develop physical symptoms or fears associated with social problems^9^. Therefore, EBPs in adolescents should be seen carefully by health professionals, parents, family members and teachers, since the early identification of these problems can improve the prognosis and adolescents’ quality of life.

It is necessary to bear in mind that sociocultural and economic contexts influence the development of EBPs. Thus, experiencing a major event such as the current crisis imposed by COVID-19 pandemic required adaptations from the whole society, especially from adolescents who face these unexpected changes in daily life with more difficulty, which can be the cause of EBP^10^.

With schools closed, restricted to the home environment, adolescents dealt daily with the fear of infection, uncertainty about the disease, frustration, boredom, inadequate information, family financial loss, family grief, and physical and social isolation. This had repercussions on various aspects of life, such as social, emotional, and behavioral ones, which can be internalized as suffering and collaborate to the development of psycho-emotional changes^11^ ^-^ ^12^.

Thus, it is essential to investigate EBP in adolescents, considering the context of the COVID-19 pandemic, and to understand the magnitude of this event in this population’s life. Such findings are essential to guide health policies and practices aimed at this population, in order to ensure harm reduction and improve its socio-emotional health. These may also encourage mobilizations to prevent, identify, welcome, refer, and treat EBP in adolescents^13^. In addition, the relevance of this study for nursing is highlighted, since the clarification of EBP in adolescents in the pandemic context will allow nurses to provide care based on the patients’ real psycho-emotional needs.

Therefore, the objectives of this study were: (1) to estimate the prevalence of emotional and behavioral problems in adolescents in the context of the COVID-19 pandemic; (2) to explore adolescents’ perception of emotional and behavioral problems identified.

## Method

### Study design 

**Figure 1:**
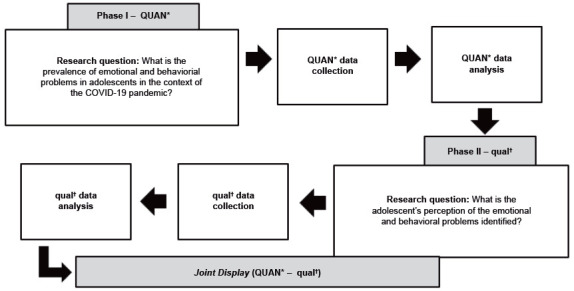
Representative diagram of the study design. Cuiabá, MT, Brazil, 2022 *QUAN = Quantitative with more weight; ^†^qual = Qualitative with less weight

This is a mixed-method study, with a two-phase explanatory sequential design. In the first phase, an observational cross-sectional study (QUAN) was carried out, and then an exploratory-descriptive (qual) was developed. The representative diagram of the investigation design is shown in Figure 1.

In the explanatory sequential design adopted in this study, the research steps fell into clear and separate but combined phases. Therefore, the quantitative data (QUAN) with more weight were collected and analyzed in a first research phase, followed by qualitative data (qual) collection and analysis, developed from the initial quantitative results (QUAN). Sequentially, data connection and interpretation (QUAN-qual) were performed. This design (Figure 1) is highly valuable to deepen (qualitatively) initial quantitative data^14^.

### Locus

The study was carried out in 21 public and four private schools in the city of Cuiabá, state of Mato Grosso, Brazil. All public and private schools in the urban area of the capital were invited to participate in the study; however, only those that authorized and supported the research were included.

### Period

Data collection for the study quantitative phase took place between April and July 2021, and the qualitative phase in July 2021.

### Population and selection criteria

The study population was composed of adolescents aged between 15 and 18 years, enrolled in secondary education at the schools included in the research.

### Participants

To estimate the expected sample in Phase I, stratified probability sampling was considered, taking into account two strata (public and private schools) in order to guarantee the representation of both. The sample calculation of the finite population was performed using Epi Info^®^ software, considering the finite population, 95% confidence level, 50% for the phenomenon, population size of 21,164 and a 5% error, resulting in a minimum expected sample of 377 individuals (287 from public schools and 90 from private schools). However, 479 adolescents joined the study (27.1% above the expected), reaching the minimum proportion estimated for each stratum.

In Phase II, 15 adolescents participated, intentionally selected, already investigated in the first phase and classified as cases of EBP, with the objective of exploring the quantitative findings of Phase I. The recruitment was ended the sample held sufficient Information Power^15^.

### Quantitative data collection and analysis

Prior to data collection, the adolescents were invited to participate in the study via messages in groups of instant messaging applications administered by schools and/or by emails directed to parents or guardians. The invitation, sent by the school management, contained information and clarifications about the research and the link to access the electronic form for quantitative data collection. The form included the participants’ identification data, such as: age, sex (male and female), education network (public and private), and the Strengths and Difficulties Questionnaire (SDQ), without impact supplement.

The SDQ is an instrument used to track mental health problems, aimed at children and adolescents, in order to assess EBP. It is translated into and validated in over 60 languages, including Brazilian Portuguese, and is easily accessed online. It is based on emotional and behavioral indicators, divided into five subscales: four for difficulties (emotional symptoms, conduct problems, hyperactivity/inattention, and peer relationship problems), and one for strengths (prosocial behavior)^16^.

The tool is composed of 25 items, five about capabilities and 20 about difficulties. Each item is scored on a scale with three answer options (not true, somewhat true and certainly true). Negative items score as: 0= not true, 1= somewhat true, and 2= certainly true; and the positives as: 2= not true, 1= somewhat true, and 0 = certainly true. For better elucidation, classification of symptoms was established as: 0= normal, 1= borderline, and 2= abnormal^16^.

For each of the five subscales, the score can vary from 0 to 10 points, with the total difficulty score being generated by the sum of the results of all subscales, except for sociability, which can vary from 0 to 40 points, since the prosocial behavior scale is not included in this score because is considered to be strength. The classification of normal, borderline, and abnormal is also established for each subscale and a total difficulty score for posterior definition of cases and non-cases of mental health problems^16^.

On the difficulty subscales, at least 80% of adolescents in the community are considered to be normal, 10% borderline, and 10% abnormal. Guidance was followed for the interpretation of the scale symptom score to define cases of mental health issues. Thus, in this study, the cases considered were adolescents identified by high or borderline scores, since the proportion of classified as “abnormal” and “borderline” was greater than 10%. However, as the “borderline” classification was below 10% and the “abnormal” was 10% for strengths, adolescents classified as borderline and normal were considered non-cases^16^.

The answers provided by the adolescents, by completing the Phase I online form, resulted in a Microsoft Excel^®^ spreadsheet. Subsequently, data processing was performed before analysis. Then, the prevalence of “normal,” “borderline,” and “abnormal” classifications was calculated for each item of the SDQ and of the EBP cases. Crude odds ratios (Orb) were also calculated using simple logistic regression for each domain of the SDQ in relation to sex. Cronbach’s alpha for the SDQ was α=0.77. All statistical analyzes were performed using STATA 16.0^®^ software, with a 5% significance level.

### Qualitative data collection and analysis

After Phase I of the research, 24 adolescents classified with EBP were selected for the focus groups (FG) in Phase II. Initially, four FGs were composed of with six adolescents each, who confirmed their participation. However, some adolescents did not attend the virtual meeting on the scheduled date and time, resulting in: five participants in the 1^st^ FG, two participants in the 2^nd^ and 3^rd^ FGs, and four participants in the 4^th^ FG. Thus, in order for the sample to hold sufficient Information Power, the 5^th^ FG was formed with addition of six more adolescents; however, only two attended.

After the 5^th^ FG, the sample reached sufficient Information Power the recruitment was ended with 15 adolescents participating in the research. Information Power is a pragmatic model that helps define the limits of participants in qualitative studies, and, for that, it considers some dimensions, namely: study objective, sample specificity, theory, dialogue quality, and analysis strategy^15^.

A meeting was held with each group in an instant messaging application, lasting an average of one and a half hours. The FG meetings aimed to collect information through participatory discussion among the respondents, gathered in the same virtual space^17^. To run the FG, a script was prepared with guiding questions, based on the Phase I results. It started with the following triggers: “How have you been feeling during the COVID-19 pandemic? Are there any feelings or behaviors that you didn’t have before and started appearing during the COVID-19 pandemic?” The script started from the general to the specific, taking into account the quantitative results, and then explores them qualitatively.

FG meetings were moderated by a research nurse with previous experience in this type of data collection, who sought to encourage everyone’s participation and the point of view of each one and the group. In addition, a nursing student with previous training was present as a reporter, and was responsible for recording the session. The key moments established in the FG were: session opening, participant presentation, clarification on the dynamics of participatory discussion, setting establishment, debate, synthesis, and session closure^17^.

The statements from the FG were fully transcribed, organized by codes, and submitted to content analysis of Bardin^18^. Participants’ statements were presented with fictitious names (chosen by the adolescents themselves), age, and the score obtained in the SDQ.

### Quantitative and qualitative data integration

**Figure 2:**
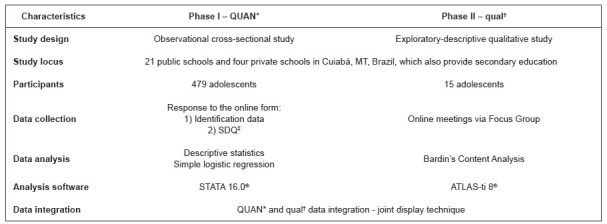
Synthesis of the study methodological characteristics. Cuiabá, MT, Brazil, 2022 *QUAN = Quantitative with more weight; ^†^which = Qualitative with less weight; ^‡^SDQ = Strengths and Difficulties Questionnaire

After quantitative and qualitative data collection and analysis, in clear and separate phases, integration was carried out through connection and joint appreciation of interpreted quantitative and qualitative results. For data integration presentation, the joint display technique was used to integrate quantitative and qualitative data during data collection, analysis and interpretation^19^. Figure 2 represents the synthesis of the methodological characteristics proposed for the study.

### Ethical aspects

This study is part of a matrix research submitted for evaluation by the Research Ethics Committee (CEP), as recommended by the National Health Council (CNS), with a favorable opinion on April 20, 2021 under No. 4.661.013. Parents and guardians of the participants under 18 years of age authorized their participation by signing the Informed Consent Term (ICF), and the adolescents signed the Assent Form (AF).

## Results

A total of 479 adolescents participated in the study, with 74.11% (n=355) of girls and 25.89% (n=124) of boys, and a mean age of 16.03 years (SD=1.01). Regarding education network, 81% (n=388) were students from public schools and 19% (n=91) from private schools.

Table 1 shows the prevalence of emotional and behavioral symptoms in adolescents, according to the SDQ items.

The prevalence of EBP was 61.17%, and there was a difference between the sexes (OR_b_=2.93; p<0.01), as can be seen in Table 2. The highest prevalence was related to peer relationship problems (54.49%) and emotional symptoms (52.40%). Only 10.23% of the adolescents showed a lack of prosocial behavior.

Among the analyzed domains, there was a difference between the sexes only in cases of emotional symptoms (OR_b_=4.39; p<0.01) and hyperactivity (OR_b_=2.26; p<0.01).

**Table 1:** Prevalence of emotional and behavioral symptoms in adolescents (n=479), according to the Strength sand Difficulties Questionnaire (SDQ) items. Cuiabá, MT, Brazil, 2022

SDQ items		n (%)
**Emotional symptoms**	**I get a lot of headaches, stomach aches or sickness**	
	Normal	207 (43.22%)
	Borderline	120 (25.05%)
	Abnormal	152 (31.73%)
	**I worry a lot**	
	Normal	62 (12.94%)
	Borderline	135 (28.18%)
	Abnormal	282 (58.87%)
	**I am often unhappy, depressed or tearful**	
	Normal	167 (34.86%)
	Borderline	123 (25.68%)
	Abnormal	189 (39.46%)
	**I am nervous in new situations. I easily lose confidence**	
	Normal	92 (19.21%)
	Borderline	125 (26.10%)
	Abnormal	262 (54.70%)
	**I have many fears, I am easily scared**	
	Normal	211 (44.05%)
	Borderline	137 (28.60%)
	Abnormal	131 (27.35%)
**Conduct problems**	**I get very angry and often lose my temper**	
	Normal	91 (19.00%)
	Borderline	169 (35.28%)
	Abnormal	219 (45.72%)
	**I usually do as I am told**	
	Normal	237 (49.48%)
	Borderline	205 (42.80%)
	Abnormal	37 (7.72%)
	**I fight a lot. I can make other people do what I want**	
	Normal	287 (59.92%)
	Borderline	137 (28.60%)
	Abnormal	55 (11.48%)
	**I am often accused of lying or cheating**	
	Normal	335 (69.94%)
	Borderline	70 (14.61%)
	Abnormal	74 (15.45%)
	**I take things that are not mine from home, school or elsewhere**	
	Normal	445 (92.90%)
	Borderline	25 (5.22%)
	Abnormal	9 (1.88%)
**Hyperactivity**	**I am restless, I cannot stay still for long**	
	Normal	240 (50.10%)
	Borderline	144 (30.06%)
	Abnormal	95 (19.83%)
	**I am constantly fidgeting or squirming**	
	Normal	129 (26.93%)
	Borderline	105 (21.92%)
	Abnormal	245 (51.15%)
	**I am easily distracted, I find it difficult to concentrate**	
	Normal	79 (16.49%)
	Borderline	151 (31.52%)
	Abnormal	249 (51.98%)
	**I think before I do things**	
	Normal	214 (44.68%)
	Borderline	209 (43.63%)
	Abnormal	56 (11.69%)
	**I finish the work I’m doing. My attention is good**	
	Normal	125 (26.10%)
	Borderline	236 (49.27%)
	Abnormal	118 (24.63%)
**Peer relationship problems**	**I would rather be alone than with children of my age**	
	Normal	137 (28.60%)
	Borderline	126 (26.30%)
	Abnormal	216 (45.09%)
	**I have one good friend or more**	
	Normal	331 (69.10%)
	Borderline	73 (15.24%)
	Abnormal	75 (15.66%)
	**Other people my age generally like me**	
	Normal	181 (37.79%)
	Borderline	214 (44.68%)
	Abnormal	84 (17.54%)
	**Other children or young people pick on me or bully me**	
	Normal	335 (69.94%)
	Borderline	93 (19.42%)
	Abnormal	51 (10.65%)
	**I get along better with adults than people my own age**	
	Normal	159 (33.19%)
	Borderline	156 (32.57%)
	Abnormal	164 (32.24%)
**Prosocial behavior**	**I try to be nice with other people. I care about their feelings**	
	Normal	359 (74.95%)
	Borderline	95 (19.83%)
	Abnormal	25 (5.22%)
	**I usually share with others, for example, CDs, games, food**	
	Normal	198 (41.34%)
	Borderline	177 (36.95%)
	Abnormal	104 (21.71%)
	**I am helpful if someone is hurt, upset or feeling ill**	
	Normal	354 (73.90%)
	Borderline	101 (21.09%)
	Abnormal	24 (5.01%)
	**I am kind to young children**	
	Normal	298 (62.21%)
	Borderline	139 (29.02%)
	Abnormal	42 (8.77%)
	**I offer to help others (parents, teachers, children)**	
	Normal	240 (50.10%)
	Borderline	165 (34.45%)
	Abnormal	74 (15.45%)

*SDQ = Strengths and Difficulties Questionnaire

**Table 2:** Prevalence and crude odds ratios (OR_b_) of emotional and behavioral problems in adolescents (n=479), according to total difficulties and its four subscales and one for strengths (prosocial behavior), general and by sex. Cuiabá, MT, Brazil, 2022

SDQ domains		Sex		Logistic Regression	
	General n (%)	Male n (%)	Female n (%)	OR_b_^†^(_95%_CI^‡^)	p-value
**Total difficulties**					
No	186 (38.83%)	72 (58.06%)	114 (32.11%)	1	
Yes	293 (61.17%)	52 (41.94%)	241 (67.89%)	2.93 (1.92; 4.46)	<0.01
Emotional symptoms					
No	228 (47.60%)	91 (73.39%)	137 (38.59%)	1	
Yes	251 (52.40%)	33 (26.61%)	218 (61.41%)	4.39 (2.79; 6.89)	<0.01
Conduct problems					
No	305 (63.67%)	88 (70.97%)	217 (61.13%)	1	
Yes	174 (36.33%)	36 (29.03%)	138 (38.87%)	1.55 (1.00; 2.42)	0.05
Hyperactivity					
No	268 (55.95%)	87 (70.16%)	181 (50.99%)	1	
Yes	211 (44.05%)	37 (29.84%)	174 (49.01%)	2.26 (1.46; 3.50)	<0.01
Peer relationship problems					
No	218 (45.51%)	65 (52.42%)	153 (43.10%)	1	
Yes	261 (54.49%)	59 (47.58%)	202 (56.90%)	1.45 (0.96; 2.19)	0.07
**Prosocial behavior**					
Yes	430 (89.77%)	108 (87.10%)	322 (90.70%)	1	
No	49 (10.23%)	16 (12.90%)	33 (9.30%)	0.69 (0.36; 1.31)	0.26

*SDQ = Strengths and Difficulties Questionnaire; ^†^OR_b_ = Crude Odds Ratio; ^‡^CI = Confidence Interval

The presentation of the integrated data (QUAN-qual) is below according to the four subscales for difficulties (emotional symptoms, conduct problems, hyperactivity and peer relationship problems) and one for strengths (prosocial behavior).

### Emotional symptoms

Among the participants, 52.40% (n=251) had emotional symptoms, and the most prevalent items in this domain were “I worry a lot” (n=282; 58.87%), and “I am nervous in new situations. I easily lose confidence” (n=262; 54.70%). Girls had 4.39 times (p<0.01) the odds of having those symptoms than boys. In the qualitative phase, the adolescents, especially girls, reported that the pandemic scenario aroused several feelings, such as anxiety, sadness, and powerlessness, among others. For them, school closures, restrictions on social interaction and concerns about the future had a considerable impact on their routines and life projects, resulting in a greater emotional suffering: […] *From the beginning of the pandemic I felt lonely, hmm, down, anxious, I kept comparing myself* [to other people]*, that’s why I talked about social media, because in the period it* [the pandemic] *appeared, I started comparing myself* […] (Apolo, 17, SDQ-Por 16). […] *when I found myself in this powerlessness situation, that there wasn’t anything I could do, you know? That destroyed me* (Pedro, 18, SDQ-Por 24). *I’m very anxious, and it got worse because of the pandemic.* […] *I start crying, then I feel bad, sad. This is awful* (Mary, 17, SDQ-Por 20).

### Conduct problems

A total of 36.33% (n=174) of adolescents were classified as having conduct problems, in which the most prevalent symptoms were “I get very angry and often lose my temper” (45.72%; n=219), and “I am often accused of lying or cheating” (15.45%; n=74). Qualitatively, the reports of impulsivity, aggressiveness, and difficulty in socializing were evident and integrate with the quantitative results: […] *after this pandemic started, I stopped talking to all my friends and started spending more time on my cell phone, and it’s hard, because the longer I spend on the cell phone, the more I get away from people* (Antônio, 15, SDQ-Por 23). *It’s just that I’m not that good at socializing, talking to others, I’m more into staying on my cell phone* […] (Jaqueline, 16, SDQ-Por 19). *I’m very rude, when I’m angry with someone I can’t help not to saying it to their face, my expression changes, when I get angry with the person, my expression changes, but at the same time it* [anger] *goes away.* […] (Júlia, 15, SDQ-Por 25).

### Hyperactivity

A total of 44.05% (n=211) of the adolescents were classified as hyperactive, and the items “I am easily distracted, I find it difficult to concentrate” (n=249; 51.98%) and “I am constantly fidgeting or squirming” (n=245; 51.15%) were the most prevalent. In the qualitative phase, adolescents revealed a lack of attention during meals, as well as during online school classes, mainly due to the use of smartphones. The smartphone is seen as a distraction that interferes with the adolescents’ attention during the development of other activities of daily living, and has impaired their ability to learn: *I can only have lunch if I’m watching something, I can’t just sit down and eat* […] (Pedro, 18, SDQ-Por 24). *I’m having trouble studying because of lack of focus* (Pedro, 18, SDQ-Por 24). […] *I can’t sit and study and do these things, I end up not paying attention to many things I have to do for getting distracted by other things, by messages, because you can’t study without being connected to the internet* (Liana, 17, SDQ-Por 19).

In the quantitative phase, it was also identified that girls had 2.26 times (p<0.01) the odds of presenting hyperactivity than boys, and they reported physical symptoms of restlessness, agitation, and anxiety in the qualitative phase: *These tics she talked about, like bouncing legs, shaking hands, biting nails, for being nervous* (Mary, 17, SDQ-Por 20). *When, sometimes, I have a crisis during the day, then the next day I sometimes get a headache, and also the tremors, like those tics of moving my legs, moving my hand, my arm, sometimes I have these tics* (Jaqueline, 16, SDQ-Por 19). 

### Peer relationship problems

Peer relationship problems were identified in 54.49% (n=261) of the adolescents, marked mainly in the quantitative phase by the items “I would rather be alone than with children of my age” (n=216; 45.09%) and “I get along better with adults than people my own age” (n=164; 32.24%). Qualitative results support that these problems increased with the COVID-19 pandemic, as it resulted in greater detachment from friends and, consequently, a greater emotional distress: *My cycle of friends changed completely after the pandemic* […] (Apolo, 17, SDQ-Por 16). […] *I distanced myself from practically everyone, school was what maintained our friendship* (Luiza, 16, SDQ-Por 23). […] *Ah I miss* [the friends]! *I do miss them, there are people who are very important to me, you know?* […] (Isis, 15, SDQ-Por 17).

### Prosocial behavior

Prosocial behavior was identified in 89.77% (n=430) of the adolescents. The items “I try to be nice with other people. I care about their feelings” (74.95% n=359) and “I am helpful is someone is hurt, upset or feeling ill (73.90%; n=354) demonstrated high prevalence of these attitudes by adolescents in the quantitative phase. Additionally, the qualitative phase highlighted the care that adolescents have with family and friends and their involvement with social issues, which were intensified during the pandemic: *At* [name of educational institution], *I’ve always participated in social movements, in projects and I wanted to be at the forefront of that thing, of something to change, you know* […] *We bought basic food baskets for the students, because there were many in a difficult, vulnerable situation. We also helped with the issue of providing Internet access to many students* (Pedro, 18, SDQ-Por 24). *Ah, I’ve helped all my friends who were down and stuff* […] *I also support several causes, I started telling people the causes I support, teaching other people* (Apolo, 17, SDQ-Por 16).

Table 3 presents quantitative and qualitative data integration through the joint display technique.

**Table 3:** Joint display of emotional and behavioral problems among adolescents (n=479). Cuiabá, MT, Brazil, 2022

Domain/Theme	Quantitative results	Qualitative results	Integrated analysis
Emotional symptoms	251 (52.40%)	Loneliness: *“From the beginning of the pandemic I felt lonely”*	Most adolescents were classified as having emotional symptoms, which were intensified during the pandemic period, especially among girls.
		Anxiety: *“I’m very anxious, and it got worse because of the pandemic”*	
		Sadness: “*I start to cry, then I feel bad, sad”* feeling of powerlessness: *“I found myself in this powerlessness situation”*	
		Repressed feelings: *“I’d rather keep it to myself than tell people”*	
Conduct problems	174 (36.33%)	Impulsivity and aggressiveness: *“I can’t control myself at the time of fight;” “I’m very rude”*	Most of the adolescents had conduct problems. The pandemic context generated more impulsivity, aggressiveness and feelings of anger, in addition to a greater difficulty in socializing.
		Anger: *“I get angry with the person”*	
		Difficulty in socializing: *“I’m not that good at socializing;” “I avoid talking to people”*	
Hyperactivity	211 (44.05%)	Psychomotor agitation: *“bouncing legs, shaking hands, biting nails;” “like those tics of moving my legs, moving my hand”*	The prevalence of the hyperactivity symptom was high among adolescents, and the COVID-19 pandemic intensified this symptom, especially in view of the emergency remote teaching adopted in this period. Girls were the most affected by these symptoms.
		Lack of attention: *“getting distracted by other things;” “I can’t just sit down and eat;” “lack of focus”*	
		Difficulty in absorbing learning: *“I’m having trouble studying”*	
Peer relationship problems	261 (54.49%)	Distancing from friends: *“I distanced myself;” “I distanced myself form many friends;” “many friends distanced from me”*	Most of the adolescents presented conduct problems. The pandemic has impacted these relationships and symptoms, generating emotional distress for adolescents.
		Renewing friendships: *“My cycle of friends changed”*	
Prosocial behavior	430 (89.77%)	Participation in social movements: *“I’ve always participated in social movements”*	Almost all adolescents presented prosocial behavior. With pandemic outbreak, adolescents began to value actions to benefit someone else even more.
		Family cooperation: *“I help my mother take care of my sister”*	
		Socio-economic support: *“We bought basic food baskets;” “We also helped with the issue of providing Internet access to many students”*	
		Emotional support: *“I’ve helped all my friends”* Sharing knowledge: *“teaching other people”*	

## Discussion

This study identified quantitatively that most of the investigated adolescents were classified as having EBP, and girls had 2.93 (p<0.01) the odds of having those symptoms than boys. Among the EBPs, the most prevalent were peer relationship problems and emotional symptoms. In addition, among the analyzed domains, there was a difference between the sexes only in cases of emotional symptoms and hyperactivity, and girls were the most affected. The qualitative results reinforce the quantitative ones and highlight that the context of the COVID-19 pandemic influenced the development and also exacerbated EBPs, according to the adolescents’ statements.

Another piece of research was conducted in Germany during the pandemic period^20^. The German study sought to investigate children’s and young people’s perception of the increase in psychological stress, and found that about 19% of those surveyed said they felt psychologically worse due to the pandemic^20^. Thus, it can be inferred that the COVID-19 pandemic had a negative impact on the adolescents’ mental health, especially due to the domestic quarantine. A survey carried out with children and adolescents in India found a higher prevalence of EBPs among those who were in domestic quarantine compared to those who were not^21^. However, this measure was extremely necessary to control the transmission of the disease.

Most adolescents had emotional symptoms, especially girls, as shown by the quantitative results. The qualitative phase emphasizes that the adolescents noticed important emotional changes in the pandemic period. Likewise, studies carried out in China, Australia, and the United Kingdom also found that girls are more affected by negative emotional symptoms than boys^22^ ^-^ ^24^. Among them, the increase in depressive symptoms during the pandemic was associated with an increase in passive social media use and a decrease in connection with family members by phone or social media, which makes it difficult for adolescents to trust their networks for emotional support at stressful times^23^ ^,^ ^25^.

In addition, it was identified that boys practice more vigorous physical activity compared to girls, which may provide protection against negative mental health outcomes^26^. A meta-analysis study found that there was an increased prevalence of symptoms of depression and anxiety among young people (mean age 13 years), when comparing pre-pandemic to the pandemic period. In the current scenario, one out of four young people has clinically severe symptoms of depression, and one out of five has clinically severe symptoms of anxiety^27^.

The quantitative results of this study showed that adolescents, especially girls, showed hyperactivity during the pandemic period. In line with this, a UK study also used the SDQ to assess the adolescents’ mental health before and during the COVID-19 pandemic, and the researchers identified that adolescents with above-average mental health before the pandemic experienced a notable increase in hyperactivity^24^. Concern about other people contracting COVID-19, changes in daily and school routines, and lack of personal contact with friends are among the main aspects that contribute to the development of mental health problems in young people, especially in relation to girls^25^.

According to the qualitative results, given the physical isolation and school closures, adolescents had difficulty in concentrating on a certain activity and in maintaining serenity. These effects were also observed in a study carried out with parents and guardians of children and adolescents in Italy, in which 85.7% of them perceived changes in their children’s behavior during quarantine, and the most frequent symptoms were difficulty concentrating (76.6 %), boredom (52%), irritability (39%), and restlessness (38.8%)^28^.

Furthermore, the results of this study indicate that most adolescents manifested peer relationship problems, mainly due to physical distance, separation from friends, loss of school life, and intensification of family contact. These results are similar to those observed in a survey developed in the UK, in which the majority of participants reported a decline in well-being due to feeling lonely and isolated, especially at the beginning of the pandemic. For adolescents, the impossibility of seeing friends or family and the loss of important life events were a source of sadness and frustration, affecting their relationships directly^29^.

Regarding the family context, similar to the results obtained with adolescents in this research, a study developed with young Brazilian adults identified that 80% of respondents reported some type of stress with the increased family socialization during the pandemic. Among them, those that claimed to be experiencing family stress situations are the majority among those living with a greater number of people in the residence^30^, a common reality for many Brazilian families.

Studies carried out in other countries, such as Spain, Indonesia, and Bangladesh, have also detected an increase in conduct problems during the pandemic. Also, a direct association of this increase with conflicts in relationships with family members, dysfunctional parenting, break in routine, and excessive time on screens is perceived^31^ ^-^ ^33^. The qualitative results identified in this research reinforce the increase in impulsivity, aggressiveness, and difficulty in socializing during the COVID-19 pandemic.

The high prevalence of prosocial behavior evidenced in this study was also described by other researchers^24^ ^,^ ^34^. Prosociality is essential to the formation and maintenance of bonds between people living in societies. Altruistic behavior is associated with reduced aggression, improved physical and mental health, longevity, and improved well-being. However, the decision to act in a prosocial manner is influenced by the environment and the circumstances experienced^24^ ^,^ ^34^.

In this sense, the context of the COVID-19 pandemic seems to have interfered positively with the adolescents’ prosocial behavior, through care of family and friends and involvement with social issues intensified during the pandemic^35^, also identified in the qualitative analysis phase of this study.

Reinforcing these findings, the results of a Dutch survey showed that during the first three weeks of pandemic lockdown, young people aged between 10 and 20 years showed increased levels of perspective-taking and socio-cognitive vigor compared to the pre-lockdown period. In addition, stable levels of general contributions to society, social value orientation, altruism, and extreme prosociality were observed^36^.

The results of this study point to the need to implement actions to promote and recover the mental health of adolescents classified with EBP, in order to mitigate the pandemic socio-emotional impacts on this population. Thus, attention to the adolescents’ socio-emotional health should be the responsibility of both parents and guardians, as well as of health, education and State professionals, by providing various means of psychological support and allow them to overcome the difficulties faced in this period^22^.

Therefore, tracking EPBs is essential during nursing care, with mental health screening being performed to assess predisposing factors (for example, temperament, adaptation, school functioning, interactions with peers, psychosocial determinants) and mental well-being factors (through emotional and behavioral indicators^37^, as the timely identification of EBPs allows for appropriate interventions to restore a better mental health status^38^.

It is possible to note the importance of nurses working in partnership with adolescents to promote their mental health and the prevention of EBPs through the individual therapeutic project during nursing consultations, collective health actions, family support, and support for the environments they are inserted in, like school. In this sense, the promotion of socio-emotional skills, such as self-esteem, responsible decision-making, and expressive communication of emotions are essential for achieving better mental health^39^.

In this collaboration between nurses and adolescents, it is important to know the health history and possible protective factors that reduce the probability of developing EBP and that can be mobilized in the nursing care process. Among these factors are: family intimacy/closeness, positive relationship with parents, development of effective interpersonal skills with peers, the feeling of belonging, good school performance, and creation of future expectations^40^.

Thus, nursing should seek to guide the adolescents so that they become emotionally capable of adopting increasingly healthy behaviors. Training is more than providing information, it is also intervening to promote the adolescent development so that they can make a realistic assessment of the consequences of their actions and can opt for more adaptive and health-promoting behaviors^39^.

Finally, regarding the limitations of this research, the use of the cross-sectional study design in Phase I does not imply causality, and the self-report questionnaire used is susceptible to social desirability bias. Therefore, the inclusion of other sources of information in future studies may help to minimize these limitations. There were many refusals by schools, justified by the high demand generated by emergency remote teaching on management and teachers. Therefore, one believes that outside this atypical period it is possible to obtain a greater adherence from schools. In addition, there was difficulty for adolescents to adhere to the FG in Phase II of the research. In further studies, it will be possible to employ other recruitment methods and data collection techniques in order to improve participant adherence.

## Conclusion

This study found that most adolescents presented EBP, and the girls were the most affected. Furthermore, results show that emotional symptoms and problems with peer relationship problems were the most prevalent among the EBPs.

The adolescents’ perception of EBP corroborated the quantitative results, since reports such as increased loneliness, anxiety, sadness, distancing from friends, and socialization difficulties were frequent. It was evident that the COVID-19 pandemic and the period of social seclusion had a negative impact on the adolescents’ mental health. On the other hand, there was an increase in participation in social movements, family cooperation, socioeconomic and emotional support for third parties, which demonstrates that adolescents, despite experiencing many negative aspects during the pandemic, also began to value more actions in benefit of other people, recognizing them as fundamental to social well-being.

For this reason, one understands the importance of proposing and implementing actions to promote and recover the adolescents’ socio-emotional health, with the objective of reducing the harmful pandemic effects on these young people’s mental health to the minimum possible, considering the difference between sexes.
